# Fungus Hæmatodes Removed by Excision, and a Radical Cure Effected

**Published:** 1841-12

**Authors:** John Travis

**Affiliations:** Carroll County, Tennessee


ART. II.—*Fungus Hæmatodes removed by excision, and a radical cure effected.* By JOHN TRAVIS, M. D., of Carroll County, Tennessee.

Previous to giving an account of the cure of the disease which stands at the head of this article, it may not be improper to make a quotation from the works of Samuel Cooper, M. D., a member of the Royal College of Surgeons, in London:

"We are indebted to Mr. Burns, of Glasgow, for the first distinct account of this formidable disease; and the subsequent and additional particulars of the subject, by Hey, Wardrop, and others, afford a good deal of information respecting the history of the distemper. It commences with a small colorless tumor, which if soft, when not covered by an aponeurosis, but firm when situated beneath it. When the disease occupies merely the adipose, cellular membrance, upon the surface of the muscles, the tumor is not usually painful in its beginning; nor does it impede the motion of the muscles on which it is seated. But when deeply seated in the limbs, it causes pain and weakness of the part affected. Also, when it occurs in the mamma, its growth is attended with considerable pain. For a considerable time the tumor is smooth and even, but afterwards projects irregularly at one or more points, and here the skin becomes thinner and of a livid red color. The swelling has a considerable degree of elasticity, yielding to pressure and rising again, immediately when this is taken off. The sensation of a fluctuation often seems to be so manifest that the mistaken surgeon plunges a lancet into the tumor, with the intention of discharging the fluid supposed to be present. An error of this kind is generally a serious one, as a painful bleeding fungus, which rapidly acquires a very large size, shoots out of the opening, and, by the irritation and loss of blood which it occasions, soon destroys the patient. But in the natural course of the disease, openings are at length formed in the projecting parts of the swelling, and their bloody matter is discharged. Almost immediately after the formation of these apertures, a small fungus protrudes, which rapidly increases both in breadth and height, and frequently bleeds profusely. The discharge is thin and exceedingly fœtid. The integuments round the ulceration are red and tender. If the patient survives similar tumors frequently make their appearance in other situations.

"There is no remedy with which we are acquainted that seems to have the least power in checking this formidable disease; all escharotics, even undiluted *oil of vitriol*, are incapable of destroying the fungous growths as fast as they are regenerated. Nothing seems to offer a prospect of preserving life except the early and total removal of the disease with the knife. This, of course, is not always practicable on account of the situation of the tumor; when it can be done, no part of the surface, surrounding the tumor, should be left, as the disease would certainly recur."

From this brief history of a disease so formidable and herculean in its character, an attempt to perform a radical cure must appear arrogant or presumptuous; and when I was called to the case, which will presently be related, I told the patient and his friends that it was *possible* that a cure could be effected, but extremely improbable. I intimated that I would use every suitable effort to remove the disease and prolong life.

Harman Gardner, of Carroll county, Tennessee, ætat. 30, at the age of 10 years had a burn on the occiput, which was cured in the usual time and a perfect eschar formed. In the year 1832 an accidental injury was inflicted on the eschar, which caused a slight hemorrhage. He had no apprehensions from so trivial an injury, and continued to pursue his ordinary occupation. The wound, however, failed to heal after the application of various domestic remedies. At the expiration of 10 or 12 months, a fungous growth commenced; blood, of a fœtid character, continually oozing out of it in small quantities. It became very painful, and in the course of 7 or 8 months attained the size of a goose egg. It was very springy, resembling the common fungus or mushroom. By this time he was unable to labor, and could not wear a hat on his head.

He called on Dr. Hogg, of Huntingdon, who informed him that his disease was a *Fungus Hæmatodes*, and that the only means of relief was that of an operation, to which Mr. G. would not then submit. He then applied to a faith doctor, in Kentucky, who promised to cure him. After making a fair trial with the faith doctor and finding no relief, and his case assuming an alarming character, he sent for me to see him in March, 1834. I removed the large tumor with the scalpel, losing about 2 pints of blood. I applied a powder, every third day, composed of arsenious acid, opium, and sulphur. Ordered him to take a 6 gr. blue pill every other night, and gave him a decoction of the root of the laurus sassafras for his ordinary drink; diet light. He had an excellent nurse, a sister, whom I ordered to remove, with a keen instrument, every particle of fungi that might, from time to time, appear; which was done accordingly. On examination I found two more fungi, near the nape of his neck, about the size of a partridge egg. They had assumed a red livid appearance. I advised the patient to have them removed, but he would not consent, asking permission to wait a week or two. In two weeks I again visited my patient, and found the appearance of the sore favorable. The other two fungi had penetrated or ruptured the integuments, and were bleeding. I found other two tumors of the size of a pea, one on his left scapula, and the other on his neck. They had not yet caused a discoloration of the cuticle. I removed the four tumors with the knife, and gave the same prescription as above, adding an occasional anodyne.

In four months my patient was well, and has remained so ever since, without the slightest symptom of a return of the disease. He is now married, follows his ordinary labor, enjoys excellent health, and remains a citizen of Carroll county.

June 8, 1841.

